# Vertical transport in graphene-hexagonal boron nitride heterostructure devices

**DOI:** 10.1038/srep14519

**Published:** 2015-09-29

**Authors:** Samantha Bruzzone, Demetrio Logoteta, Gianluca Fiori, Giuseppe Iannaccone

**Affiliations:** 1 Dipartimento di Ingegneria dell’Informazione, Università di Pisa. Via G. Caruso 16, 56122 Pisa, Italy

## Abstract

Research in graphene-based electronics is recently focusing on devices based on vertical heterostructures of two-dimensional materials. Here we use density functional theory and multiscale simulations to investigate the tunneling properties of single- and double-barrier structures with graphene and few-layer hexagonal boron nitride (h-BN) or hexagonal boron carbon nitride (h-BC_2_N). We find that tunneling through a single barrier exhibit a weak dependence on energy. We also show that in double barriers separated by a graphene layer we do not observe resonant tunneling, but a significant increase of the tunneling probability with respect to a single barrier of thickness equal to the sum of the two barriers. This is due to the fact that the graphene layer acts as an effective phase randomizer, suppressing resonant tunneling and effectively letting a double-barrier structure behave as two single-barriers in series. Finally, we use multiscale simulations to reproduce a current-voltage characteristics resembling that of a resonant tunneling diode, that has been experimentally observed in single barrier structure. The peak current is obtained when there is perfect matching between the densities of states of the cathode and anode graphene regions.

Vertical heterostructures of two-dimensional materials are the subject of intense investigation for the possibility they offer to engineer and taylor specific electrical and optical characteristics^1^.

In graphene-based electronics, transistors based on vertical heterostructures are studied because they can achieve large current modulation, if large bandgap layers are included that can effectively block current in the off state. Therefore, a few transistor concepts based on vertical transport have been proposed^2^,^3^ and experimentally demonstrated^4^,^5^,^6^ in recent years.

Vertical heterostructures of two-dimensional materials are qualitatively different from the well known heterostructures based on the III-V and II-VI materials systems. Indeed, adjacent layers can have very different Hamiltonians and transverse energy-dispersion relations. This aspect can lead to the emergence of peculiar transport properties.

The choice of the materials stacked with graphene is crucial, since the deposition and growth of graphene on a dielectric substrate can significantly alter its electronic properties and suppress^7^,^8^ the extremely high mobility observed in suspendend samples^9^,^10^,^11^,^12^.

Hexagonal boron nitride (h-BN) is considered a promising dielectric^13^,^14^,^15^,^16^, having a honeycomb lattice closely matching that of graphene^17^, and only weakly interacting with deposited monolayer^14^,^18^ or bilayer graphene^19^.

In this paper, we report some peculiar properties of vertical transport through single- and double-barrier heterostructures of graphene and h-BN or h-BC_2_N layers, that we have studied by means of density functional theory.

In detail, we will show that tunneling through a single insulating barrier has a small dependence on energy in a large portion of the bandgap (Section II), and that a graphene layer between two tunneling barriers leads to a suppression of resonant tunneling and to an increase of the average transmission (Section III).

Such properties are relevant for the operation of vertical electron devices and are not observed in better known heterostructures based on III-V and II-VI materials systems.

Finally, following a multiscale approach, and leveraging the results of ab initio simulations, we have developed a tight-binding model for graphene/h-BN/graphene and graphene/h-BC_2_N/graphene heterostructures. In this way we are able to reproduce—with a full quantum simulation—experimental results^20^ on a gated single-barrier device exhibiting a current-voltage characteristic resembling that of resonant tunneling device. We are able to confirm that the current peak occurs when perfect matching is obtained between the densities of states of the cathode and anode graphene regions.

## Transport properties of a single h-BN barrier

**Figure 1:**
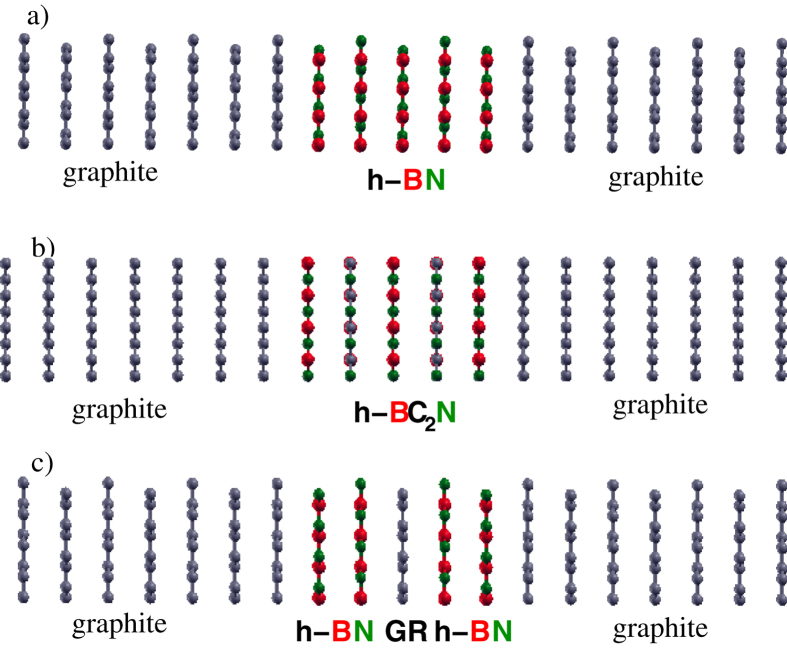
Side view of the supercell used to represent the scattering region corresponding to (**a**) graphite/5(BN)/graphite; (**b**) graphite/5(BC_2_N)/graphite; (**c**) graphite/2(BN)/graphene/2(BN)/graphite.

**Figure 2:**
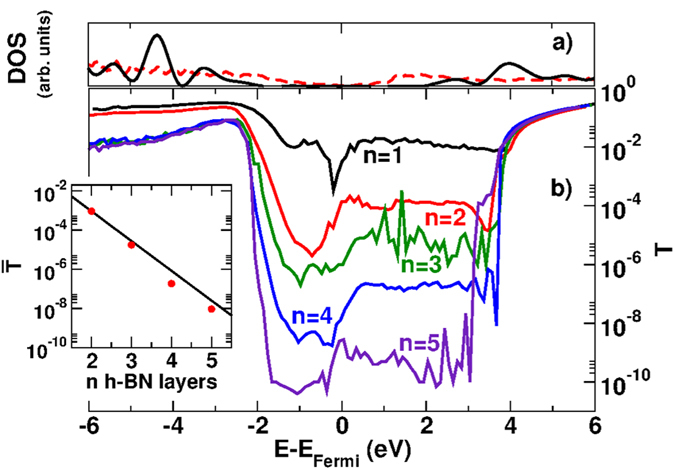
Top: Density of states of bulk h-BN (solid black line) and graphite (dashed red line). Bottom: Tunneling probability as a function of the incident energy for a single h-BN barrier of *n* atomic layers between graphite leads. *E*_Fermi_ is the intrinsic Fermi energy.

The transmission probability of a single h-BN barrier, with a number of layers *n* ranging from 1 to 5 (Fig. 1) is plotted in Fig. 2 as a function of energy. In the tunneling regime *T* decays exponentially as *n* increases^4^. This is shown in the inset of Fig. 2, in which the average values of the transmission 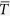 in the energy range 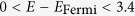 eV are plotted as a function of *n* together with the fitting exponential 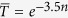.

**Figure 3:**
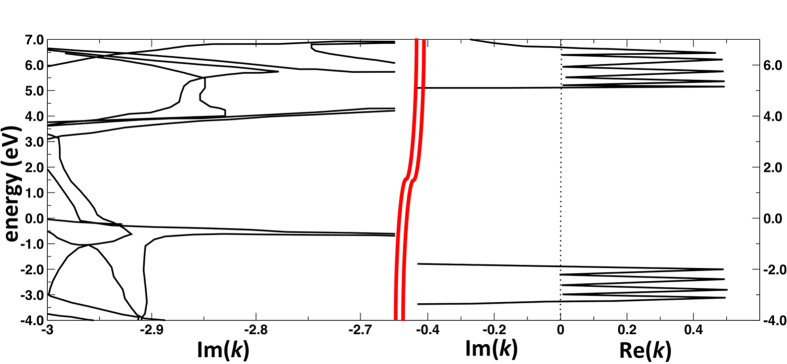
Complex band structure for h-BN bulk.

More remarkably, for a given number of single h-BN layers, the transmission probability is weakly dependent on energy in a range of about 3 eV above the Fermi level. This behavior can be explained by inspecting the complex band structure of bulk h-BN as a function of the longitudinal wave vector 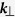 (Fig. 3). Since propagating states in graphite are characterized by wave vectors close to the 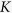 point, we set in Fig. 3 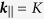. We notice that, in the **above-mentioned** energy range, the imaginary 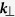 in the barrier is almost independent of energy. As a direct consequence, since 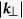 is the inverse decay length, the tunnel probability itself is only weakly dependent on energy.

We also notice that for energy *E* below the valence band edge of h-BN or above the conduction band edge 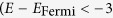 eV and 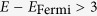 eV in Fig. 2, respectively), i.e. in a classically allowed region, the transmission probability is significantly smaller than unity. We ascribe this effect to the interruption of perfect lattice periodicity at the interface between graphite and the barrier, which leads to backscattering of the Bloch states incoming from the electrodes.

**Figure 4:**
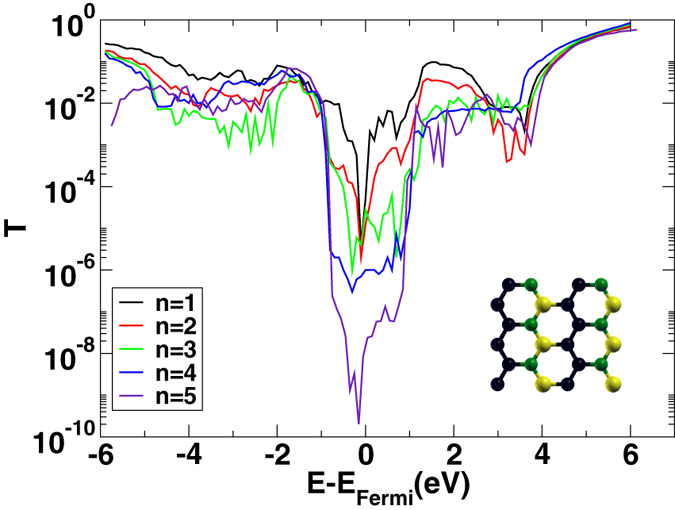
Transmission probability as a function of energy for a single h-BC_2_N barrier of *n* atomic layers between graphite leads. The inset shows the atomic structure of the considered BC_2_N isomer.

In Fig. 4, the transmission probability as a function of energy is shown for a single barrier of h-BC_2_N of *n* atomic layers. The transmission probability has an analogous behavior as in the case of h-BN both as a function of energy and of the number of layers *n*.

### Transport properties of double barriers

The systems we consider consist of two identical barriers separated by one, two, or three graphene layers. For the sake of clarity, each double barrier (DB) system is identified by means of a set of three integers (*l, m, n*), denoting the number of insulating layers on the left (*l*), the number of graphene layers (*m*) in the central region, and the number of insulating layers on the right (*n*). Analogously, hereinafter we refer to the single barrier systems with the acronym SB, followed by the number of insulating layer.

There are two noteworthy aspects in transport through double barriers that are not typically seen in different materials systems, such as for example III-V semiconductor heterostructures.

**Figure 5:**
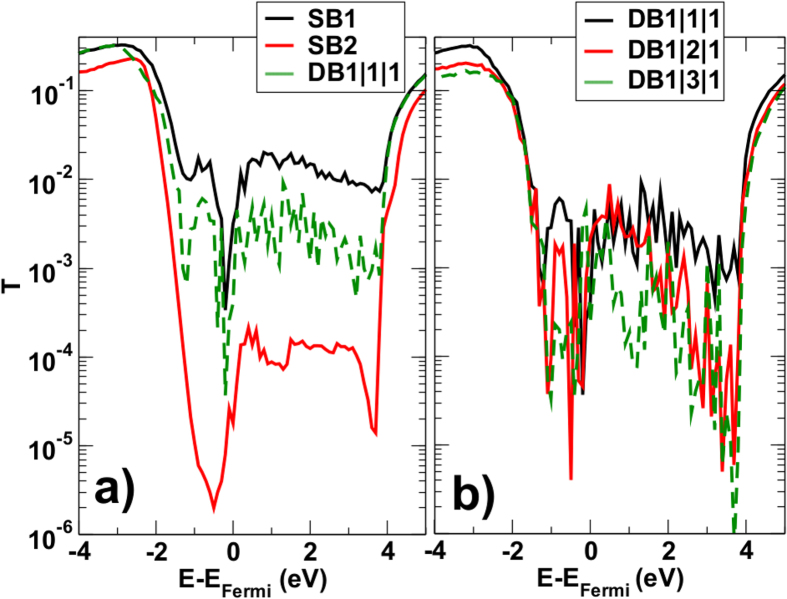
Transmission probability as a function of the energy for different systems; (**a**) SB(1) (solid black line), SB(2) (solid red line), DB(1, 1, 1) (dashed green line). (**b**) DB(1, 1, 1) (solid black line), DB(1, 2, 1) (dashed red line), DB(1, 3, 1) (dotted green line).

First, from Fig. 5(a) we observe that the insertion of a single graphene sheet has a noticeable effect of transport: the transmission probability obtained for a triple layer consisting of a graphene layer inserted between two h-BN sheets (DB(1, 1, 1)) is notably higher than that obtained for a single barrier consisting of two layer of h-BN (SB(2)). Much smaller variations are observed in Fig. 5(b), in which we compare the behavior of the transmission probability for the DB(1, 1, 1) system with those obtained by considering bilayer and three-layer graphene in between the barriers.

**Figure 6:**
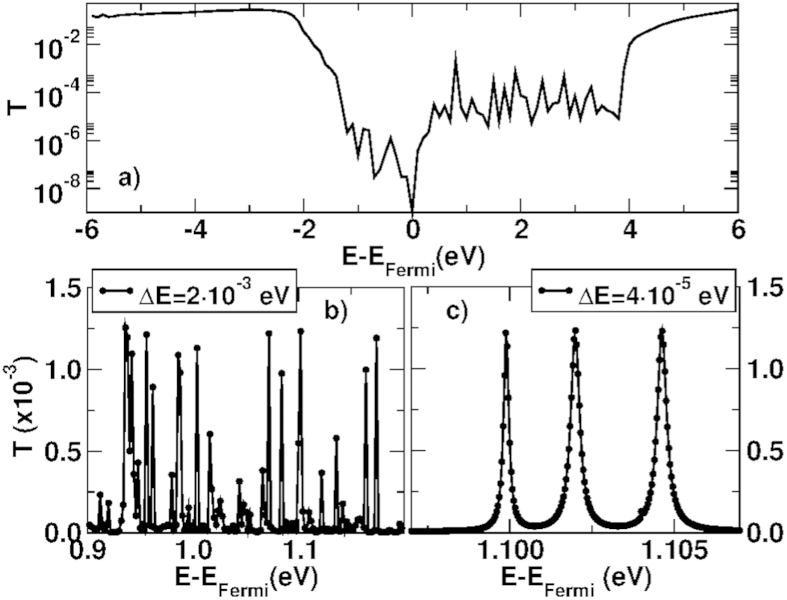
(**a**) Transmission probability as a function of the energy for DB(2, 1, 2); (**b,c**) Transmission probability as a function of energy for DB(2, 1, 2) system with step sampling 2 × 10^−3^ eV and 4 × 10^−5^ eV.

The second remarkable aspect is that in the double barrier structures we observe no resonant tunneling (Fig. 6a). In order to exclude the occurrence of very narrow resonances, we have resolved the transmission fluctuations by considering different energy steps (i.e., 2 × 10^−4^ eV and 4 × 10^−5^ eV, shown in Fig. 6b,c, respectively). The peaks of the transmission that we have been able to observe are at most of the order of 10^−3^.

We can explain both aspects by observing that the Hamiltonians of the graphene layer and of the insulator are very different, leading to very different energy-disperson relations in each trasversal plane. Therefore, the Hamiltonian of the whole double-barrier structure cannot be decoupled in the sum of a longitudinal and a transversal component. In our case, for each 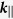 the phase accumulated by the wave function in the quantum well is very different and (only apparently) random. On the other hand, when the Hamiltonian can be decoupled, the phase accumulated by the wave function in the quantum well is independent of 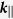, and depends only on the longitudinal energy.

**Figure 7:**
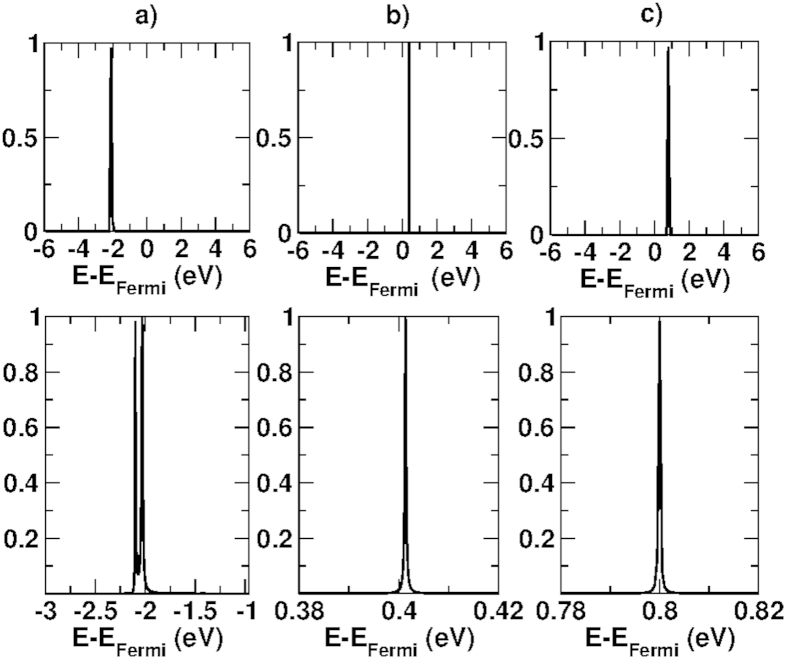
Transmission probability 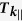 of the double barrier system DB(2, 1, 2) as a function of energy for selected 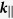: (**a**) (0.28000;0.28867); (**b**) (0.31000;0.32909); (**c**) (0.28000;0.33486). Units of 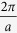 with a = 2.503 Å. Pictures at the bottom are a zoom of the transmission coefficient at the top.

This effect is confirmed if we look at the transmission probability 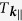 for a single 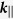 as a function of *E*, shown in Fig. 7. As can be seen, clear Fabry-Perot resonances up to unity are present. However, when we perform the sum in ((1)) all peaks occur at very different energies and are completely averaged out, so that we do not see resonant peaks anymore, but only an average increase of transmission with respect to the case in which the intermediate graphene layer is not present.

We can interpret this effect by saying that the graphene layer has an *effective phase randomizing* role. Let us stress the fact that transport is fully coherent, and that phase coherence is conserved, therefore we need to highlight that phase randomization is only “effective”.

### Multiscale simulation of single barrier heterostructure with resonant-tunneling-like behavior

It has been predicted^21^,^22^ and experimentally demonstrated^20^ that tunneling transport through single barrier graphene/insulator/graphene heterostructure can exhibit pronounced “resonant” features.

Let us stress the fact that here we are not discussing a resonant-tunneling mechanism, which requires tunneling through quasi-bound states like in a Fabry-Perot cavity, but of a different mechanism which provides a current-voltage characteristics similar to that of resonant tunneling devices, with a local current peak followed by a negative differential resistance region and a further current increase.

**Figure 8:**
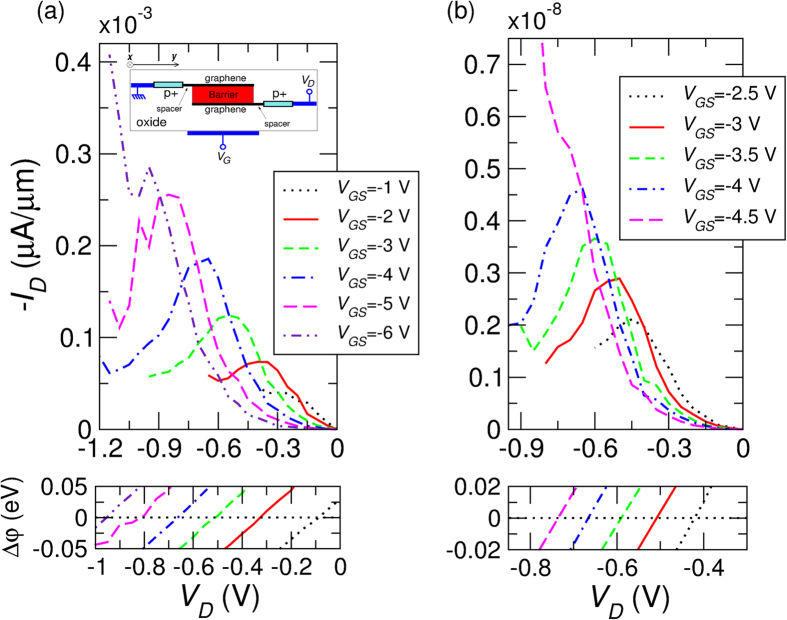
(**a**) Top panel: *I*_*D*_-*V*_*D*_ characteristic of the simulated graphene/(3-layer h-BN)/graphene heterostructure for different value of the voltage *V*_*G*_ applied to the back gate. Bottom panel: difference as a function of *V*_*G*_ between the electrostatic potential in the upper and lower graphene layer, evaluated at the *y* coordinate midway between the left and right contact. Inset: Simulation geometry of the considered gated graphene/h-BN/graphene and graphene/h-BC_2_N/graphene heterostructures. (**b**) Same as the panels (**a**), for the case of a graphene/(5-layer h-BN)/graphene heterostructure.

In this section we investigate this transport mechanism. We perform fully quantum transport simulations of gated graphene/h-BN/graphene and graphene/h-BC_2_N/graphene heterostructures, according to the simulation geometry illustrated in the inset of Fig. 8(a). The length of the region over which the graphene layers overlap is *L* = 10 nm, in line with the scaling requirements for the next generation transistors^23^. Undoped spacers of 2 nm have been considered between the drain and source contacts and the tunneling region, in order to obtain a more uniform potential in the overlap region and to inhibit in this way the thermionic component of the current. A back gate, partly ovelapping with the spacers and separated from the bottom graphene sheet by a 4 nm-thick HfO_2_ layer, electrostatically dopes the top and bottom graphene layers.

Graphene and the barrier materials have been modeled with a semiempirical nearest-neighbor tight-binding approximation^3^. The in-plane and vertical hopping parameters and the onsite energies for h-BN and h-BC_2_N have been extracted by fitting the corresponding DFT data for the transmission coefficient. The graphene lattice of the top layer is rotationally aligned with that of the bottom layer. Bloch boundary conditions have been imposed in the transversal *x* direction^3^. Simulations have been performed with the NanoTCAD ViDES package^24^. The transmission has been computed in the approximation of ballistic transport by solving self—consistently the Poisson and the Schrödinger equation with open boundary conditions at the graphene contacts. Current has been obtained from transmission by means of the Landauer formula^25^.

Figure 8(a) shows the results obtained for a heterostructure with a barrier consisting of 3 atomic layers of h-BN. The drain-to-source current *I*_*D*_ per unit length is shown as a function of the drain voltage *V*_*D*_ and for different values of the back gate voltage *V*_*G*_ in the *V*_*D*_ range in which the tunneling component of the current in predominant.

As expected, the current peak occurs for the values of *V*_*D*_ for which the Dirac point of the top and bottom graphene layers align (see bottom panel of Fig. 8(a)). It is therefore not a resonant tunneling effect, but the best match between the states of the cathode and anode graphene regions.

The width of the peak reflects the uncertainty 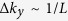 on the *y* component of the wave vector imposed by the finite size of the tunneling region in the *y* direction. Accordingly, each peak develops over the *V*_*D*_ interval in which the difference between the Dirac point in the top and bottom layer is 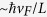, where 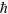 is the reduced Plank constant and *v*_*F*_ is the Fermi velocity in graphene 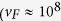 cm/s).

When 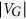 increases, a larger *V*_*D*_ is required to align the Dirac point in the graphene layers and to achieve the matching condition. As a consequence, by increasing 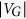, the difference between the Fermi levels in the top and bottom layer at resonance increases and a larger tunnel current flows, since a larger number of states is available for tunneling. The peak-to-valley ratio (PVR) increases approximately linearly with 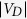, reaching a maximum of ~2.5, and then it is suppressed by the onset of the thermionic regime.

In Fig. 8(b) the results obtained by considering a 5-layers h-BN barrier are shown. We observe no significant variation in the behavior of *I*_*D*_-*V*_*D*_ characteristics and the achievable PVR, except the smaller absolute current.

**Figure 9:**
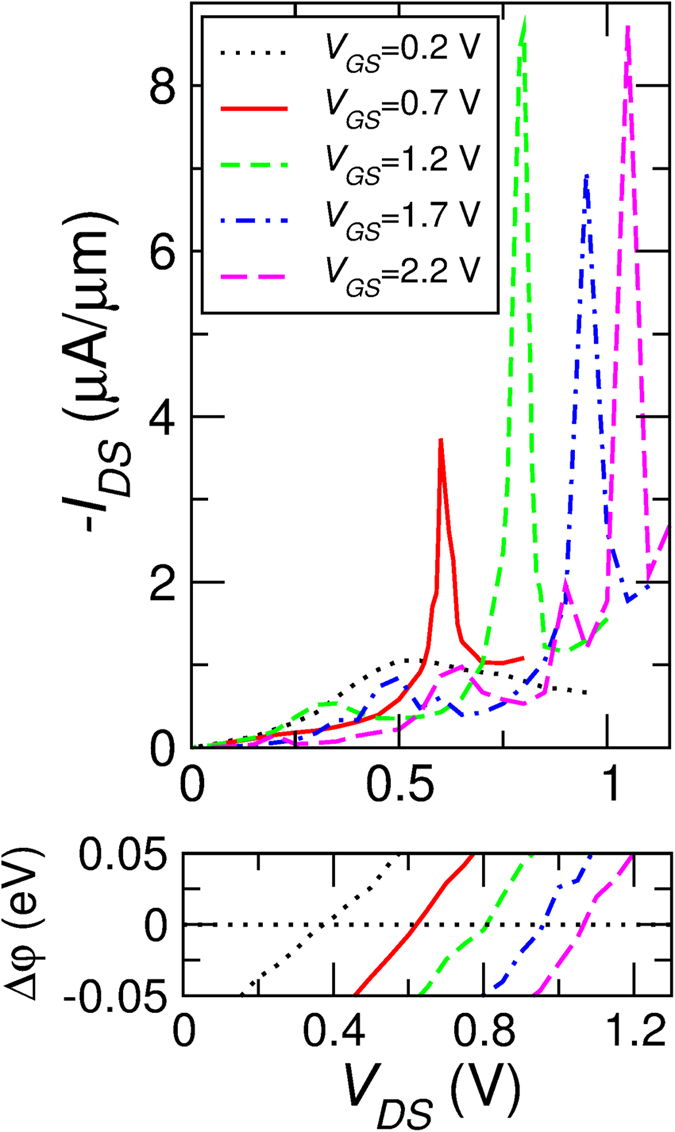
Same as Fig. 8, for the case of a graphene/(5-layer h-BC_2_N)/graphene heterostructure.

Finally, we discuss the case in which the 5-layers h-BN barrier is replaced with a 5-layer h-BC_2_N barrier (Fig. 9). In this case, in order to reduce the thermionic component of the current and observe clear peaks, we extended the length of the spacers to 9 nm and proportionally increased the length of the back gate. Consistently with the increase of the length *L*_*g*_ of the graphene layers, we observe narrower and higher resonant peaks^21^, as compared to the previous results. The smaller peaks arising on the side of the main ones occur at values of *V*_*D*_ for which 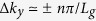, i.e. corresponding to the lateral maxima of the spectrum of the rectangular well-like confining potential. The larger obtained value of the PVR for this structure is ~8.

## Conclusion

In this paper we have provided an accurate investigation of the tunneling properties of vertical h-BN/graphene and h-BC_2_N/graphene heterostructures by means of ab-initio and multiscale simulations. Our findings are relevant to a better understanding of the operation of recently proposed electron devices based on vertical graphene-based heterostructures^3^,^5^,^6^,^13^, and to research on off-plane quantum transport in graphene.

We have shown that a set of remarkable behaviors emerge, not observed in the better known heterostructures based on III-V and II-VI materials systems. Such behaviors are due to the very different Hamiltonians and energy-dispersion relations between adjacent layers, and to the fact the the total system Hamiltonian cannot be decoupled in a longitudinal and a transversal component.

In single barrier structures we have found that the transmission probability is exponentially dependent on the number of layers, and is already strongly suppressed by a single layer of h-BN or h-BC_2_N. On the other hand, our results show that the tunneling probability is only slightly dependent on energy in the band gap, as an effect of the peculiar band structure of h-BN and h-BC_2_N for imaginary wave vectors.

In double barrier structures, obtained by intercalating h-BN and h-BC_2_N with few layer graphene, we have shown that resonant tunneling does not occur. We only observe an increase of average trasmission with respect to the corresponding single barrier systems obtained by removing graphene. Both effects are explained by the non-separability of the Hamiltonian of the complete structure, that we have also interpreted in terms of an “effective phase randomization” due to the intermediate graphene layer.

Using a multiscale approach, we have then simulated gated graphene/h-BN/graphene and graphene/h-BC_2_N/graphene heterostructures. We have shown that a large peak-to-valley ratio (of up to 10) in the current-voltage characteristics can be observed at room temperature. The peak is not due to a resonant-tunneling behavior, but to the perfect matching of the density of states of the cathode and anode graphene regions.

Further study is required to better understand vertical transport in cases in which heterostructures are not lattice-matched, or misalignment and dissipation mechanisms are present.

## Method

The single barrier structure we consider consist of up to five h-BN or h-BC_2_N atomic layers stacked between semi-infinite graphite leads and arranged according to ref. 26, as shown in Fig. 1. The double barrier structures consist of two or four h-BN or h-BC_2_N layers separated by up to three graphene layers and connected to semi-infinite graphite leads. To ensure the continuity of the charge density at the interface between each lead and the scattering region, we include on both sides of the latter at least four atomic layers of graphite.

Ab-initio computations have been performed by means of Quantum Espresso^27^, using a plane-wave basis set with the local density approximation (LDA)^28^,^29^,^30^,^31^,^32^. The plane wave cut-off energy is 35 Ry and the kinetic energy cutoff is 300 Ry for the charge density. The Brillouin zone has been sampled using a 30 × 30 × 1 Monkhorst—Pack grid.

It has been shown^32^ that LDA provides a reliable description of the geometry and the electronic structure in the presence of weak interactions between h-BN layers or between h-BN and graphene layers. On the other hand, the LDA representation for the exchange-correlation potential cannot correctly describe the excited states in organic systems and leads to underestimate the energy gap. However, as confirmed by other works on this subject^33^, this approximation does not affect our conclusions.

The van der Waals interaction anchors the h-BN and graphite layers at the interlayer distance observed in experiments^34^,^35^. Therefore, we have optimized the geometry of the total system with the van der Waals interaction-corrected density functional (vdW-DF), based on first principles without empirical corrections, as implemented in the Quantum Espresso code^36^. The adopted exchange-correlation energy functional includes three terms: the exchange part of the PBE functional^37^, the correlation part of the standard LDA and the non-local correlation part, which allows to represent the dispersive interaction incorporating effective many body density response.

We describe the transport properties in the framework of the Landauer formalism^25^,^38^. The ballistic transmission *T* as a function of the energy *E* is evaluated as
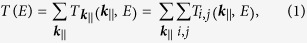
where 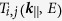 is the probability that an electron with energy *E* and transversal momentum 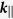 incoming from the *i*-th Bloch state is transmitted to the outgoing *j*-th state of the other contact, and 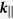 belongs to the two—dimensional Brillouin zone of the supercell. 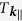 is the sum of *T*_*i,j*_ over all incoming and outgoing states. Sums run on both spins.

Transmission probabilities have been calculated with the PWCOND^39^ module of Quantum Espresso.

## Additional Information

**How to cite this article**: Bruzzone, S. *et al.* Vertical transport in graphene-hexagonal boron nitride heterostructure devices. *Sci. Rep.* **5**, 14519; doi: 10.1038/srep14519 (2015).
